# Implementing Signature Neural Networks with Spiking Neurons

**DOI:** 10.3389/fncom.2016.00132

**Published:** 2016-12-20

**Authors:** José Luis Carrillo-Medina, Roberto Latorre

**Affiliations:** ^1^Departamento de Eléctrica y Electrónica, Universidad de las Fuerzas Armadas - ESPESangolquí, Ecuador; ^2^Grupo de Neurocomputación Biológica, Dpto. de Ingeniería Informática, Escuela Politécnica Superior, Universidad Autónoma de MadridMadrid, Spain

**Keywords:** bioinspired ANNs, neural signatures, subcellular plasticity, multicoding, local contextualization, signature neural network, spiking neuron

## Abstract

*Spiking Neural Networks* constitute the most promising approach to develop realistic Artificial Neural Networks (ANNs). Unlike traditional firing rate-based paradigms, information coding in spiking models is based on the precise timing of individual spikes. It has been demonstrated that spiking ANNs can be successfully and efficiently applied to multiple realistic problems solvable with traditional strategies (e.g., data classification or pattern recognition). In recent years, major breakthroughs in neuroscience research have discovered new relevant computational principles in different living neural systems. Could ANNs benefit from some of these recent findings providing novel elements of inspiration? This is an intriguing question for the research community and the development of spiking ANNs including novel bio-inspired information coding and processing strategies is gaining attention. From this perspective, in this work, we adapt the core concepts of the recently proposed *Signature Neural Network* paradigm—i.e., neural signatures to identify each unit in the network, local information contextualization during the processing, and multicoding strategies for information propagation regarding the origin and the content of the data—to be employed in a spiking neural network. To the best of our knowledge, none of these mechanisms have been used yet in the context of ANNs of spiking neurons. This paper provides a proof-of-concept for their applicability in such networks. Computer simulations show that a simple network model like the discussed here exhibits complex self-organizing properties. The combination of multiple simultaneous encoding schemes allows the network to generate coexisting spatio-temporal patterns of activity encoding information in different spatio-temporal spaces. As a function of the network and/or intra-unit parameters shaping the corresponding encoding modality, different forms of competition among the evoked patterns can emerge even in the absence of inhibitory connections. These parameters also modulate the memory capabilities of the network. The dynamical modes observed in the different informational dimensions in a given moment are independent and they only depend on the parameters shaping the information processing in this dimension. In view of these results, we argue that plasticity mechanisms inside individual cells and multicoding strategies can provide additional computational properties to spiking neural networks, which could enhance their capacity and performance in a wide variety of real-world tasks.

## 1. Introduction

Biological neural circuits are powerful computational systems that efficiently process a great amount of data in real time with extensive plasticity capabilities. This makes the nervous system a source of inspiration when designing engineered tools. In this sense, many Artificial Neural Network (ANN) paradigms mimicking the computational principles performed by living neural systems have been developed to solve real-world problems (Michie et al., 1994; Bishop, 1995). Nevertheless, the bio-inspiration in most cases is limited to a knowledge about neural information processing that was available more than 60 years ago. A challenge in ANN research is related to incorporate novel bio-inspired information coding and processing strategies to the network design since they can contribute to enhance the network capacity to perform a given task (Rumbell et al., 2014).

Information coding in the nervous system is mainly based on the generation, propagation, and processing of action potentials or *spikes* (Bialek et al., 1991; Kandel et al., 1991; Rieke et al., 1999). Most of the neural computation is driven by these events. The classical view of neural coding emphasizes the importance of information carried by the rate at which neurons discharge action potentials. However, experimental evidence indicates that living neural systems use many different information coding strategies (Rabinovich et al., 2006b; Middleton et al., 2011), which greatly enhances their processing capacity as compared to the classical view. In this scenario, temporal coding emerges as a strategy commonly used by neural systems, emphasizing that, unlike (or in addition to) the firing rate paradigm, neural information may be carried by precise individual spike timings (e.g., see Mainen and Sejnowski, 1995; Lestienne, 1996; Diesmann et al., 1999; Reinagel and Reid, 2002).

Traditional ANN paradigms are mostly based on highly simplified information processing mechanisms derived from the neural coding classical view. However, the growing experimental evidence of the importance of temporal code to explain neural computation gave rise to the *Spiking Neural Networks*, nowadays considered the third generation of ANNs (Gerstner, 1995; Maass, 1997b). In the two previous generations, neuron models employ threshold gates and activation functions, such as sigmoid functions, to propagate analog values to their neighbors. In contrast, spiking neurons communicate and encode information using discrete spikes (Gerstner et al., 1993; Deco and Schürmann, 1998; Maass and Bishop, 2001; Gerstner and Kistler, 2002; Bohte, 2004; Brette et al., 2007; Ponulak and Kasinski, 2011). This allows spiking neural networks to solve computational tasks using a firing-rate based strategy as their analog counterparts (O'Connor et al., 2013; Diehl et al., 2015; Esser et al., 2016), but discrete spiking activity provides additional dimensions for information coding (e.g., time, frequency or phase), which makes ANN of spiking neurons a promising approach for solving complex computational tasks. Theoretical efforts try to illustrate that computing and modeling with these networks may be biologically plausible and computationally efficient (Maass, 1997a; Izhikevich, 2004; VanRullen et al., 2005; Cessac et al., 2010). It has been shown that spiking neural networks are at least as computationally powerful as traditional ANN paradigms (Maass, 1996, 1997a; Natschläger and Ruf, 1998; Ruf and Schmitt, 1998). In applied engineering, spiking ANNs have been successfully used in different practical applications, such as motor control, odor recognition, image classification, or spatial navigation between others (see Ponulak and Kasinski, 2011, for an overview).

Although they are closer to their biological counterparts, most ANN paradigms of spiking neurons do not include relevant computational principles experimentally and theoretically studied in the nervous system. For instance, most neuro-inspired paradigms consider network elements as indistinguishable units; they only implement synaptic learning based on adjusting the synaptic weights (Bohte et al., 2002b; Kube et al., 2008; Ponulak and Kasinski, 2011); and individual units are considered integrators that integrate synaptic input over time until a given threshold is reached. Experimental evidence demonstrates that neural computation does not only include synaptic integration and synaptic plasticity, but also *subcellular plasticity*, i.e., intra-unit mechanisms that allow a neuron to tune its intrinsic dynamics and shape the computation of its output response as a function of the incoming information (Zhang and Linden, 2003; Turrigiano and Nelson, 2004; Davis, 2006; Turrigiano, 2007). Likewise, it is commonly considered that the information arriving to a neuron is encoded through a single code, e.g., the rate or the precise timing of spikes, when the need for several simultaneous codes (*multicoding*) in the nervous system seems to be apparent (Latorre et al., 2006; Kayser et al., 2009; Panzeri et al., 2010). Living cells receive many inputs from different sources and send their output to different neurons too. An effective way to improve communications is combining multiple encoding modalities in the same signal. Not all the readers have to be interested in the same modality at the same time, specially when we talk about multifunctional networks. This kind of information processing requires of local information discrimination/contextualization mechanisms that allow a neuron to process the multiple simultaneous codes in its input signals one by one or simultaneously in order to perform different tasks. Subcellular plasticity emerges as a highly relevant strategy to perform this context-dependent information processing.

*Signature Neural Networks* represent a novel self-organizing bio-inspired ANN paradigm that incorporates some of these concepts (Latorre et al., 2011). Behind this ANN paradigm, there are three main ideas. (1) Each neuron of the network has a signature that allows its unequivocal identification by the rest of the cells. (2) The neuron outputs are signed with the neural signature. Therefore, there are multiple codes in a message regarding the origin and the content of the information. (3) The single neuron discriminates the incoming information and performs a distinct processing as a function of the multiple codes in the network. Nevertheless, in spite of being inspired in a precise temporal structure, signature neural networks are non-spiking ANN. The main goal of this work is to assess whether the information coding and processing strategies proposed by the signature neural network paradigm are plausible for spiking networks. With this aim, we morph the core concepts of the existing non-spiking paradigm to build an ANN of spiking neurons.

Bursting activity consists of series of high-frequency spikes that alternate with quiescent periods with only subthreshold activity (Izhikevich, 2006). This is particularly suitable to implement multicoding, since it involves the presence of at least two different time scales that can serve to encode distinct informational aspects. It has been also suggested that the burst length or the number of spikes in a burst can be used by living neurons to encode information (Kepecs and Lisman, 2003, 2004). Information can also be encoded in the intraburst firing pattern. In the bursting activity of the leech heartbeat control circuit, the temporal structure of the first spikes in the burst allows predicting the length and number of spikes of the burst (Campos et al., 2007). Another relevant temporal structure within the burst is the intraburst neural signature, in which the signature neural network paradigm is inspired. *Intraburst neural signatures* are very precise and cell-specific spike timings experimentally observed in the bursting activity of cells of different vertebrates and invertebrates living neural circuits (Szücs et al., 2003, 2005; Garcia et al., 2005; Zeck and Masland, 2007; Brochini et al., 2011). In central pattern generators (CPGs), they depend on the synaptic organization of the network (Latorre et al., 2002; Rodríguez et al., 2002; Szücs et al., 2003). These precise temporal structures coexist in the neural signals with relevant information encoded with other encoding modalities. Their possible functional meaning for the neurons that belong to the same or to other neural system is still an open question. Model simulations of CPG circuits (Latorre et al., 2004, 2006, 2007) point out that they can have important implications for the understanding of the origin of the CPG rhythms, the fast and fine tuning to modulation and the signaling mechanisms to other interconnected systems (other CPGs or muscles that the CPG controls). These modeling results have shown that cell-specific intraburst spike timing can be part of a multicoding strategy of bursting neurons. The readers of these signals may be able to read these characteristic firing patterns to perform different tasks in response to the multifunctional signals from each CPG cell.

In the context of ANN, bursting activity has been labeled as a “non-standard” behavior (Kampakis, 2013). However, taking into account the previous considerations, the individual units of the proposed network have bursting behavior. We argue that the additional dimensions to encode information provided by bursting activity can significantly increase the computational power of a spiking network. In particular, here we consider two encoding schemes in the bursting signals: a rhythmic encoding modality, in which information is carried by the bursting frequency; and a spike-timing encoding modality; in which information is carried by specific intraburst spike patterns. Each individual neuron has a characteristic intraburst neural signature that uses to sign its output signals in the spike-timing encoding dimension. Finally, the model incorporates intra-unit history-dependent processing rules to compute the response in the spike-timing encoding dimension as a function of previous incoming signals. This local contextualization mechanism can be considered a particular case of subcellular plasticity. The idea behind this network design is transforming different stimuli and/or different relevant aspects of the inputs into different coexisting spatio-temporal spaces that encode information in a distributed network form.

The analysis of the emerging collective dynamics and the self-organizing properties of the network discussed in this paper points out that novel bio-inspired processing strategies could enhance the spiking ANNs capacity and performance. In particular, we provide a proof-of-concept that combining multiple encoding modalities in the network allows transforming incoming data into different spatio-temporal spaces, from which different aspects of the data, including their source, could be exploited one by one or globally. Different collective processing strategies can be implemented in each information dimension only by tuning the synaptic or intra-unit parameters, which facilitates parallelism and multifunctionality in the network. All these features would potentially increase the computational power of spiking ANNs and their ability to model complex high-dimensional processes.

## 2. Models and methods

### 2.1. Network model

Signature neural networks use neural fingerprints to identify each individual unit of the ensemble (Latorre et al., 2011). For the spiking network proposed here, we take inspiration from the CPG circuits and use interspike interval signatures to achieve this feature. Thus, the fingerprint of a neuron (*n*_*i*_) is a cell-specific intraburst spike timing distribution described as the sequence *S*_*i*_ = {*ISI*_1_, *ISI*_2_, …, *ISI*_*n*_}, where *ISI*_*n*_ represents interspike intervals between consecutive spikes within the same burst. The timing of the last spikes in the bursting activity of the pyloric CPG cells varies from one burst to another; while the first spikes in the burst are highly reliable (Elson et al., 1999; Varona et al., 2001a,b) and contain the neural signature (Szücs et al., 2003, 2005). Mimicking this behavior, we consider two parts in a burst. The first part is used to sign the output messages and contains the signature of the emitter neuron (*S*_*i*_). The spike timings of the second part of the burst are given by a preferred output pattern (*P*_*i*_ = {*t*_0_ = 0, *t*_1_, *t*_2_, …, *t*_*N*_}) that changes dynamically as a result of the single neuron plasticity (see Section 2.1.3).

Spiking-bursting activity allows the simultaneous propagation of different units of information throughout the network (multicoding). Therefore, different spatio-temporal spaces can be simultaneously used to globally encode and store information. In the network discussed in this paper, we consider two coexisting units of information in each neural signal: the bursting frequency and the neural fingerprints included within the burst. In the first dimension, the network must generate and coordinate spatio-temporal patterns of propagating transient bursting activity (rhythmic encoding modality). To achieve this, we impose two constraints (Wiedemann and Lüthi, 2003; Tabak et al., 2010): (i) predominance of excitatory synapses and (ii) a refractory period in each neuron following hyperexcitation. Information processing in the second dimension is based on the emission and recognition of specific neural signatures (Tristán et al., 2004; Carrillo-Medina and Latorre, 2015), i.e., information in this dimension propagates encoded in a spike-timing modality. An intra-unit contextualization mechanism drives the signature emission and recognition processes. This does not only allow us to illustrate a novel information processing strategy in the context of spiking neural networks, but also the dynamical richness that subcellular plasticity can provide to these networks.

#### 2.1.1. Neuron spontaneous dynamics

Many spiking models generate output bursts depending on the parameter settings and/or the input stimuli (e.g., the models by Hindmarsh and Rose, 1984, Komendantov and Kononenko, 1996, or Liu et al., 1998 that we have previously used to investigate the functional meaning of neural signatures). However, simulations show that the neural signatures in these models mainly depends on the network connectivity (Latorre et al., 2002) and, to our knowledge, none of the existing spiking models displays an adaptive fingerprint as required by our study. A possible alternative to this issue is using the mechanism described in Marin et al. (2014) to tune neuron busting models and produce neural signatures equivalent to those observed in living cells. However, the generation of realistic signatures is out of the scope of this proof-of-concept.

**Figure 1:**
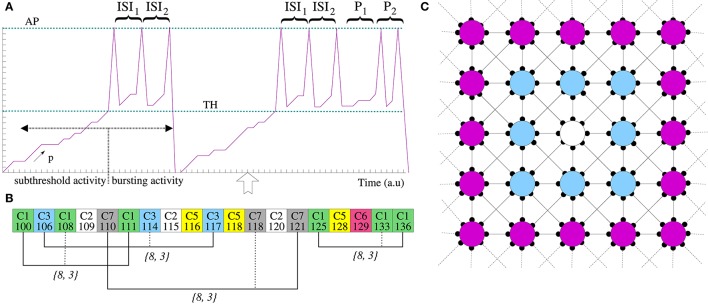
**(A)** Schematic representation of the stochastic neuron model (see main text for details). *S* = {*ISI*_1_, *ISI*_2_} and *P* = {*P*_1_, *P*_2_} denote the neuron signature and the preferred output pattern, respectively. Note that the intraburst firing pattern is different in the first and the second burst. This is because, as **(B)** illustrates, the neuron recognizes a signature at the time step pointed by the arrow and intra-unit plasticity changes the neuron response in the spike-timing encoding modality. **(B)** Example of signature recognition. For each incoming spike, the local informational context keeps track of the corresponding input channel and spike timing (e.g., C1-100 means that at time step 100 a spikes arrived to the neuron through channel C1). This transient memory provides an intra-unit contextualization mechanism to the single neuron. For example, if the arrow in **(A)** corresponds to time step 136 and an input spike arrives through channel C1, the neuron can contextualize this spike and determine that the signature {8, 3} have been received four times in the recent history. If this value is greater than the learning threshold (*L*_*i*_), the neuron recognizes this signature and, consequently, modulates its output firing pattern as illustrated in the second burst of **(A)**. **(C)** Network topology. Each neuron is directly connected to its eight nearest neighbors with periodic boundary conditions. Then, neighbors of the white unit are the blue neurons.

To describe the individual behavior of each unit, we define a stochastic model operating in a discrete event framework. The neuron activity is considered as a discrete variable and characterized in time by *V*(*t*), its “membrane potential.” Figure 1A illustrates schematically the neuron spontaneous dynamics. Our model neuron integrates and processes the information received through its different input channels (synaptic integration), adapts its firing pattern to the incoming information (intra-unit plasticity), and generates a coherent signed output signal. During subthreshold activity, the spontaneous evolution of the neuron activity is determined by the probability *p*—the transit probability of the internal state per time step. When the membrane potential of a neuron *n*_*i*_ reaches the firing threshold (*TH*), this generates a sequence of spikes (not a single spike). The temporal distribution of spikes within the response burst is given by a firing sequence composed of concatenating the signature (*S*_*i*_) and the preferred output spike pattern (*P*_*i*_) of the neuron. Then, the stochastic dynamics of a single neuron depends on the temporal evolution of the neuron activity and whether it is under (subthreshold activity) or over (spiking-bursting activity) the firing threshold. Formally:

- During subthreshold activity (*V*_*i*_(*t*) < *TH*):
  (1) $$\begin{array}{l} V_{i}\left(t+1\right)=\{\begin{array}{ll} V_{i}(t)+I_{syn}+1 & \text{with probability }p\\ V_{i}(t)+I_{syn} & \text{otherwise} \end{array} \end{array}$$
  where *I*_*syn*_ is the synaptic input (Equation 3) and *p* the transit probability of the internal state per time step.
- During the generation of the burst (*V*_*i*_(*t*) > = *TH*):
  (2) $$\begin{array}{l} V_{i}(t+1)=\{\begin{array}{ll} AP & \text{if }t=t_{1}+t_{n}\\ TH+1 & \text{if }t=t_{1}+t_{n}+1,\;\;\forall n\neq N\\ 0 & \text{if }t=t_{1}+t_{N}+1\\ V_{i}(t)+1 & \text{otherwise with probability }p\\ V_{i}(t) & \text{otherwise} \end{array} \end{array}$$
  where *N* is the number of spikes in the firing sequence (*S*_*i*_ + *P*_*i*_), *t*_*n*_ denotes the timing of the nth spike in this sequence, (i.e., *t*_1_ corresponds to the initial timing of the burst) and being *AP* the peak membrane potential to generate a spike. Note, that during the burst generation synaptic input (*I*_*syn*_) is not taken into account (cf. Equation 1 and 2). After generating a burst, neurons have a refractory period of *RP* time steps during which *V*_*i*_(*t*) = 0. Then, subthreshold dynamics starts again.

#### 2.1.2. Synaptic input

Synaptic input arrives to a neuron through two kind of input channels: connections with other neurons and an external channel to introduce external stimulation into the network. Each neuron in the network is connected to its eight nearest neighbors (Figure 1C) with periodic boundary conditions. As in every spiking neural network, neurons communicate with each other through the generation and propagation of spikes. Then, the interchange rule is defined by:

(3) $$\begin{array}{l} I_{syn}=g_{e}\cdot pulse_{e}+\underset{j}{\sum }g_{ji}\cdot pulse_{j} \end{array}$$

where *g*_*e*_ defines the weight of the external stimulus, *pulse*_*e*_ is 1 when an action potential is delivered through the external channel and 0 otherwise; and, similarly, *g*_*ji*_ is the weight of the connection between neurons *n*_*j*_ and *n*_*i*_ and *pulse*_*j*_ is 1 when *V*_*j*_(*t* − 1) = *AP* and 0 otherwise. Note that Equation 3 does not apply neither during the generation of a burst (Equation 2) nor during the refractory period, i.e., in these situations synaptic input is not considered.

It is important to highlight that in this paper we do not discuss synaptic learning (see Section 4). This implies that *g*_*ji*_ is constant for all the synapses and, consequently, the neighborhood of every neuron does not change.

#### 2.1.3. Intra-unit plasticity

Incorporating subcellular plasticity to a neuron model implies that a mechanism inside the cell allows tuning the neuron dynamics to incoming signals and/or to particular processing states. We consider here a history-dependent contextualization mechanism driving the spike-timing encoding modality. This intra-unit contextualization modulates the preferred output pattern as a function of previous incoming spike patterns.

As in the non-spiking signature neural network paradigm, to implement local contextualization, each individual neuron uses a transient memory, called *local informational context*. The local informational context keeps track of the information received during a time window of *M*_*i*_ time units, providing a history-dependent contextualization mechanism to the single neuron processing. In the case of our spiking network, for each incoming spike, the neuron stores in its local context the joint information about the input channel and the spike timing (Figure 1B). In this way, different intra-unit plasticity rules can be defined to take into consideration the input spike timings. In particular, the following rule can be used to recognize specific neural signatures:

- when a spike arrives to a target unit, this checks whether the spike pattern received though the corresponding input channel appears in its local informational context so many times as a given learning threshold, *L*_*i*_. If so, the receptor recognizes this fingerprint, which implies that the preferred output pattern is overwritten with the recognized fingerprint.

Figures 1A,B illustrate how intra-unit plasticity tunes the output firing pattern in response to the fingerprint recognition. During the generation of the first burst in the time series, the neuron does not recognize any signature. Therefore, there is not a preferred output pattern and the burst only contains the signature of the neuron (*S* = {*ISI*_1_, *ISI*_2_}). At time step 136 (pointed by the arrow), an spike arrives through channel C1. The neuron can use its local informational context (Figure 1B) to contextualize this spike. In our case, this means to identify the incoming pattern through this channel (in this case {8, 3}) and to determine that this fingerprint has been received four times from time step 100. Then, assuming that the learning threshold is *L*_*i*_ = 4, the neuron's preferred output spike pattern changes due to the recognition of the signature *S*′ = {8, 3}. As a consequence, the intraburst firing pattern of the second burst in the time series varies to encode additional information (in the example, the sequence *P* = {*P*_1_ = 8, *P*_2_ = 3}). The neuron emits the new preferred output pattern until a new fingerprint is recognized or until the recognized fingerprint appears less than *L*_*i*_ times in the local informational context (keep in mind that this is transient memory). Note, that intra-unit plasticity can be used to compute different aspect of the output signal as a function of the local contextualization, not only the spiking firing pattern. For instance, a particular cell could increase/decrease its level of activity or generate an output spike in response to specific incoming patterns independently of the synaptic weight.

During the input processing, channels are checked randomly in each iteration. In this way, when the target neuron recognizes multiple signatures in the same iteration, the last processed prevails over the others. Plasticity rule does not apply during the generation of a burst—i.e., once the neuron starts firing, the output spike pattern cannot change.

### 2.2. Analysis methods

#### 2.2.1. Rhythmic encoding modality

To illustrate the spatio-temporal patterns generated in the bursting informational dimension, we generate activity movies representing the membrane potential evolving dynamics. In these movies, the evolution in time of the activity of a given unit (*V*_*i*_(*t*)) is represented with a color scale. Regions with the same color have synchronous behavior. Red corresponds to neurons with a membrane potential over the firing threshold (*V*_*i*_(*t*) > *TH*), i.e., they are generating a burst. Intermediate colors between blue and red, represent subthreshold activity. The cooler the color, the lower the level of activity.

Spatio-temporal patterns of spiking or spiking-bursting activity in one dimensional signals are usually detected and analyzed by means of spectral methods. However, in higher dimensions, the coefficients produced by the multidimensional Fourier transform are hard to interpret. On the other hand, wavelet-based techniques have proven to be useful tools for signal analysis (Stollnitz et al., 1996; Mallat, 1999). Unlike the Fourier transform coefficients, the wavelet transform coefficients are determined both by a resolution component and a time (or space) component and, therefore, they represent the resolution content at a given portion of the original signal. Thus, to quantitatively characterize the bursting rhythmic activity in our network, we perform a wavelet-based analysis.

In particular, we use the same discrete wavelet transform (DWT) analysis employed in Latorre et al. (2013a) to characterize the global network dynamics of a model of the inferior olive. The method consists in considering the spiking-bursting spatio-temporal patterns produced by the network as sequences of images evolving in time. As a first step in the characterization, a two-dimensional basis is generated by direct Cartesian product of the one-dimensional Haar basis (Stollnitz et al., 1996). Then, the two dimensional non-standard DWT is calculated for each frame of network activity. The idea behind this characterization method is that the number of wavelet coefficients in a given frame, *C*(*t*), provides an estimation of the complexity of the image corresponding to the spatio-temporal pattern at time *t*. A low number of coefficients means that the image is smooth or is composed of smooth components. In contrast, a high number of coefficients corresponds to complex images. In this way, the DWT analysis transforms the multidimensional spiking-bursting activity in the network, *V*_*i*_(*t*), into a one dimensional signal, *C*(*t*). This signal provides an useful characterization of the bursting dynamics in which both the frequency and the spatial complexity can be discussed. From the frequency perspective, a simple visual inspection of the evolution of *C*(*t*) allows to detect the presence of different rhythmic patterns in the network. Furthermore, these rhythms can now be studied by means of the one dimensional Fourier transform. From the spatial complexity of the patterns, very high values of *C*(*t*) correspond to almost random behavior of every neuron, with no patterns present; intermediate high values indicate the presence of complex spatial structures in the patterns; while completely synchronized networks produce a small number of coefficients. Note, that *C*(*t*) ranges between 0 and the number of neurons in the network.

#### 2.2.2. Spike-timing encoding modality

The spike-timing encoding is related to the spreading of specific intraburst spike patterns through the network and the synchronization mechanisms that allow a group of neurons to recognize and emit the same signature at a given moment (Tristán et al., 2004; Carrillo-Medina and Latorre, 2015). To graphically illustrate the dynamic spatial organization of the spike patterns within the network, we generate activity movies representing the fingerprint-based evolving dynamics (e.g., see **Figure 5**). Each point in the 50 × 50 square represents with a color code the neural signature recognized by a given neuron within the network at a given moment. In this manner, neurons with the same color recognize the same signature. White color identifies the units that do not recognize any fingerprint.

To quantitatively analyze this encoding strategy, we compute the evolution of the number of neurons that recognize and emit each individual signature per time unit. This measure provides an estimation over time of the level of activity in the network related to each signature.

## 3. Results

We have conducted experiments in which multiple datasets are presented to regular networks with different parameters. Independently of the network size and the number of neighbors per neuron, it is possible to find a broad range of synaptic weights and neuron parameters allowing the network to simultaneously encode information in the rhythmic and the spike-timing modality. However, the emerging phenomena that we describe here can be more easily illustrated in autonomous networks with a low level of bursting activity, since in these cases, the spatio-temporal activity in the different dimensions arises due to external stimulation. In autonomous networks, i.e., networks not receiving external input, the level of bursting activity depends on the transit probability of the internal state (*p*), the firing threshold (*TH*), and the duration of the refactory period (*RP*). These parameters modulate the ratio of bursts produced by an isolated neuron. The greater the value of the stochastic probability *p*, the higher the mean bursting frequency. Similarly, the bursting frequency also grows with low values of *TH* and *RP*.

Thus, in the following sections, we focus on neurons where *p* = 0.05, *TH* = 50, *RP* = 50, and *AP* = 200 (units are dimensionless). Note, that *AP*—the peak membrane potential to generate a spike—has no influence on information processing, the only requirement is being greater than *TH*. We discuss results of square-shaped networks of 50 × 50 of such units, with periodic boundary conditions and where each unit is connected through an excitatory synapse (*g*_*ji*_ = 1) to its eight nearest neighbors as shown Figure 1C. External stimuli consist of tonic spiking signals at a given frequency introduced into a randomly chosen cell during a give time period. The neural signature of every neuron has six spikes, with all the ISIs in the range 2–12 (dimensionless). These signatures are randomly generated and assigned at the beginning of the simulation. The rest of parameters are specified in the corresponding experiment description. *V*_*i*_(0) is chosen randomly between 0 and 40 *a*.*u*. for all neurons in the network.

### 3.1. Rhythmic encoding modality

**Figure 2:**
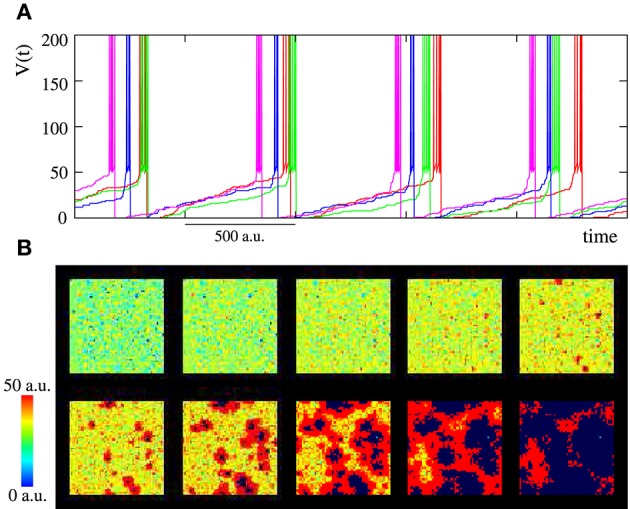
**(A)** Activity time series of four randomly chosen close neurons in an autonomous network with *M*_*i*_ = 400 and *L*_*i*_ = 4. Units are dimensionless. Due to the synaptic excitation, the generation of a burst in a given unit propagates to the surrounding units. **(B)** Spatio-temporal patterns of spontaneous activity observed in the network of the top panel. The patterns consist of propagating wave fronts of spiking-bursting activity. Sequences develop in time from left to right and from top to bottom with a time interval between frames of 33 *a*.*u*.

The degree of synchrony among the membrane potential of the neurons constituting the network characterizes the global spiking-bursting activity in the network. For fixed values of *p* and *TH*, the degree of synchrony varies as a function of the synaptic transmission strength among neurons (i.e., *g*_*ji*_ in Equation 3). For small values, each neuron fires nearly independently. As synaptic weights grow, the degree of synchrony increases because the generation of a burst in a given unit sequentially propagates to its neighbors and so on (Figure 2). The higher synchrony occurs in networks with combinations of firing thresholds and excitatory synapses that allow a target neuron to reach the firing threshold when it receives a burst (*g*_*ji*_ · *#spikes*_*in*_*burst* ≥ *TH*). However, as we mention above, here we are interested in autonomous with a low level of bursting activity.

Depending on the synaptic parameters, burst propagation provides autonomous networks the ability to generate well-defined spatio-temporal patterns in the form of propagating wave fronts of transient spiking-bursting activity. Note that local contextualization modulates intraburst firing patterns, but it has not any influence on burst timings. To illustrate these spatio-temporal patterns, we generate activity movies representing the membrane potential evolving dynamics (see Section 2.2.1 for details). As representative example of the spontaneous collective bursting rhythms generated by the network, bottom panel in Figure 2 displays snapshots of the activity movie of the network shown in the top panel.

**Figure 3:**
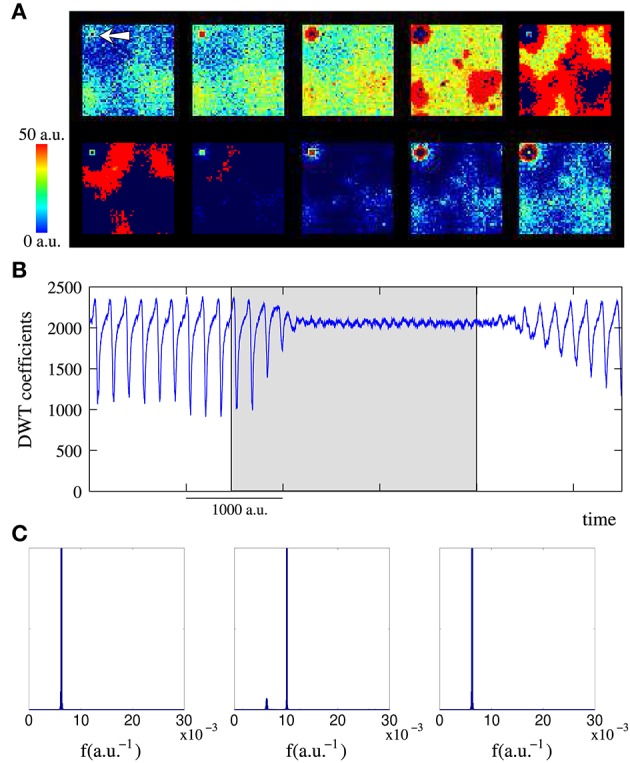
**(A)** Snapshots of an activity movie illustrating the spiking-bursting spatio-temporal patterns generated by the network of Figure 2 (*M*_*i*_ = 400 *a*.*u*. and *L*_*i*_ = 4) when a tonic input with a period of 100 time units between consecutive spikes is introduced into a single neuron (arrow in the first frame points to the neuron that receives incoming stimulus). Sequences develop in time from left to right and from top to bottom. The time interval between frames is 33 *a*.*u*. The stimulated unit increases its bursting frequency due to the external stimulation, and this generates new spatio-temporal patterns of transient spiking-bursting activity from this unit. **(B)** Characterization with the DWT coefficients of the activity of the network in the top panel: first without stimuli (snapshots in Figure 2B belong to this period), then when the selected neuron receives the incoming data (grayed area identifies the period while the input is active), and finally without any input again. **(C)** Normalized power spectra of the wavelet analysis for the three periods. Left: without stimuli. Middle: during the stimulation. Right: when the stimulation is over and after the reverberation period. Power spectra are calculated using time series of 500,000 time units. The DWT analysis demonstrates that the global network dynamics changes when data are introduced into the network. It also shows that the network is a dynamical working memory of spiking-bursting rhythms, since the network dynamics generated in response to data onset reverberates after the input is retired.

The spontaneous generation of transient spatio-temporal patterns of spiking or spiking-bursting activity is a feature with relevant functional implications observed in different living neural media. However, we are interested in the network response to stimuli. Therefore, from the encoding perspective, the most interesting feature of the network, appearing even in networks with a small synaptic transmission among neurons, is its ability to develop dynamical patterns of spiking-bursting activity in response to data onset. These patterns allow the network to encode information using the frequency of different bursting rhythms induced by stimuli. To illustrate how the network of Figure 2 encodes a single input using this spatio-temporal space, Figure 3A shows snapshots of its collective spiking-bursting dynamics when a unit in the left-top corner receives an external tonic spiking signal. When the stimulus is introduced into the neuron, its firing frequency increases. Then, the spiking-bursting activity originated in the stimulated neuron propagates to the surrounding units because of excitation. Thus, this neuron becomes the origin of a new rhythm that coexists with those generated spontaneously by the network (if any).

The DWT analysis (Section 2.2.1) corroborates the rhythm encoding in the network transient spiking-bursting dynamics. Changes in the collective spiking-bursting dynamics in response to data onset are reflected in a change in the evolution of the DWT coefficients whose shape characterizes the spiking-bursting activity of the network. As an example, Figure 3B illustrates how the collective dynamics of the network in Figure 3A changes when data are introduced into the stimulated unit. Initially, no data is present and the network spontaneously generates spatio-temporal patterns as the ones shown in the bottom panel of Figure 2. In this situation, the DWT coefficients oscillate with a nearly homogeneous frequency capturing the spontaneous spiking-bursting rhythm. The spontaneous rhythm frequency depends on the stochastic probability *p* and can be estimated by means of the Fourier transform of the wavelet analysis of the network activity. For instance, in the network of Figures 2, 3, the spontaneous rhythm frequency is around 6.2·10^−3^ (see frequency peak in Figure 3C, left). On the other hand, the oscillation of the DWT coefficients between a high and an intermediate value indicates, respectively, the nearly independent neuron behavior during subthreshold activity and a high transient synchronization in the network during the spreading of the spiking-bursting wave fronts. Then, the external stimulus is introduced into the network during a given time interval (grayed area). At this point, the network collective dynamics stepwise changes. A first remarkable change in the evolution of DWT coefficients is observed in the oscillation amplitude. Now, the DWT coefficients tend to oscillate around two high values. This change points out the complex spatial structure of the new emerging dynamics. Not obtaining low or intermediate values in the DWT analysis during the stimulation period indicates that, in this network, the propagation of the wave fronts originated in the stimulated unit does not imply a complete transient synchronization in the whole ensemble. Another relevant change in the DWT coefficients during the stimulation is a frequency increase (cf. left and middle power spectra in Figure 3C), pointing out that the rhythm evoked by the stimulus prevails over the spontaneous rhythm (6.2·10^−3^ vs. 10·10^−3^ *a*.*u*.^−1^). The frequency of the spiking-bursting rhythms evoked by external stimulation depends on the frequency of the input, since the stimulated neuron follows the stimulus. These changes indicate that the network has encoded the incoming information in a characteristic spiking-bursting rhythm. Finally, no input is present again and the network recovers the spiking-bursting autonomous activity (cf. Figure 3C, right). The DWT analysis indicates that the stimuli-evoked rhythms can reverberate for long periods after data onset. This implies that the network behaves as a working memory in the spiking-bursting spatio-temporal space. For each network configuration, the mean reverberation period of the rhythms encoding different inputs is nearly the same, i.e., the memory capability of the network in this information dimension is independent of the data and only depends on the synaptic parameters.

**Figure 4:**
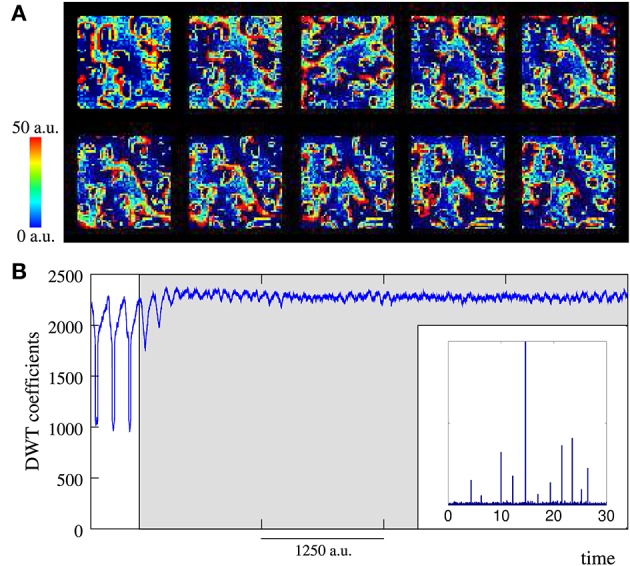
**Figure equivalent to Figure 3 but when the network (***M***_***i***_ = 400 ***a***.***u***. and ***L***_***i***_ = 4) receives 10 inputs**. **(A)** Sequences develop in time from left to right and from top to bottom. The time interval between frames is 33 *a*.*u*. In response to data onset, the network starts generating 10 different coexisting rhythms encoding incoming information. **(B)** The different spiking-bursting rhythms encoded within the network are captured by the DWT analysis. While external inputs are present, the oscillation frequency of the DWT coefficients is not homogeneous (see inset), which reveals the coexistence of the different rhythms. Inset shows the normalized power spectrum of the wavelet analysis of a time series of 500,000 time units while the 10 external stimuli are present. The number of coefficients increase (cf. Figure 3B) denotes the increase in the spatial complexity of the patterns.

The emerging collective dynamics analysis in networks that receive multiple tonic stimuli with different frequencies indicate that spatio-temporal patterns of spiking-bursting activity allow the network to encode information using several coexisting and coordinated rhythms. Top panel in Figure 4 displays an example of the complex spatial organization of the patterns generated by a network receiving 10 different inputs. The snapshots clearly show the increased complexity of the patterns, since, now, the network organizes clusters of neurons oscillating at different frequencies (cf. top panel in Figure 3). Each of the unit receiving external data becomes the source of a rhythm that propagates through the network competing with the rhythms encoding other inputs. As we show above, while an input is active, the corresponding rhythm survives in the network. Therefore, when more than one stimulus is present, the competition among the input-evoked spiking-bursting rhythms is a winnerless competition. Note that there is no inhibition in the network nor subcellular plasticity rules limiting the spiking-bursting activity. Winnerless competition allows the encoding of multiple coexisting spiking-bursting rhythms. This competition dynamics is captured by the DWT analysis (bottom panel in Figure 4). When multiple data are introduced into the network, the number of DWT coefficients remains high with a non-homogeneous oscillation frequency. This reveals the complex spatial structure of the patterns and, on the other hand, the coexistence of multiple spiking-bursting rhythms within the network.

We have previously shown that the spiking-bursting rhythms evoked by a single stimulus reverberate for a while when the stimulation is over. The reverberation period drastically increases when the network receives multiple stimuli. The greater the number of external inputs, the greater the number of sources of spiking-bursting activity. This translates into a higher spiking-bursting activity in the network and explains the increasing reverberation period. Depending on the synaptic strength and the value of *p*, in this situation, the network even becomes a long-term memory of spiking-bursting rhythms. We would like to emphasize that the rhythms that survive for longer periods in short-term memories or the ones that persistently reverberate in long-term memories are not always the higher frequency stimuli-evoked rhythms nor the rhythms encoding the last data presented to the network.

### 3.2. Spike-timing encoding modality

One of the major characteristics of the proposed network is the intra-unit contextualization of input signals, responsible of the spike-timing encoding modality. In this section, we study the complex collective dynamics induced by this intra-unit information processing strategy. These emerging collective dynamics can give us important clues about the underlying computational properties of the network.

As expected, the ability of an individual unit to recognize specific fingerprints varies as a function of the intra-unit parameters shaping local contextualization, i.e., the maximum size of the local informational context (*M*_*i*_) and the fingerprint learning threshold (*L*_*i*_). Depending on the value of these parameters, specific intraburst firing patterns can propagate through autonomous networks. However, the more interesting phenomena from the information processing viewpoint are related to the mechanisms that allow the network to generate and organize spatio-temporal patterns in response to data onset. Therefore, we focus our attention on networks in which the signature recognition does not occur without external stimuli. When these networks receive incoming data, they aid the study of the information encoding in the fingerprint-based spatio-temporal space by analyzing how the signatures of the stimulated units propagate throughout the network.

**Figure 5:**
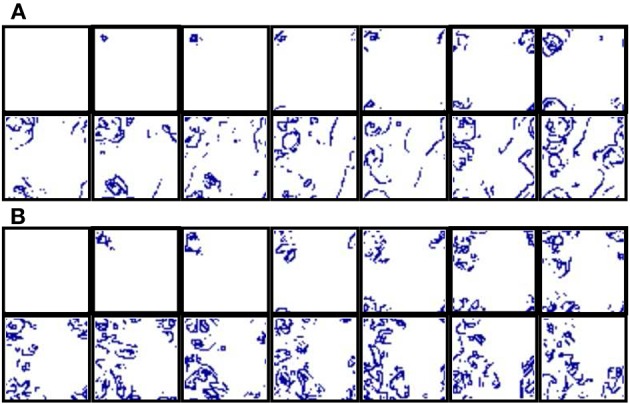
**Snapshots of two representative activity movies illustrating the fingerprint-based encoding mechanism**. **(A)** *M*_*i*_ = 500 *a*.*u*. and *L*_*i*_ = 5. **(B)** *M*_*i*_ = 400 *a*.*u*. and *L*_*i*_ = 4. Sequences develop in time from left to right and from top to bottom. The time interval between frames is 1000 *a*.*u*. Note that the propagation of the fingerprint-based spatio-temporal patterns is slower than the corresponding spiking-bursting rhythms (cf. bottom panel and Figure 3). The color code identifies neurons recognizing the same signature, being white color used for neurons that do not recognize any signature. The first frame in each sequence indicates that, in the absence of stimuli, neural signatures do not propagate in these networks. When the external stimulus is introduced into a neuron located in the left-top corner (second frame in both panels), new collective dynamics emerge and the network organizes transient spatio-temporal patterns of activity related to the propagation of the signature of the stimulated unit (blue regions). Note that this is the only signature that travels throughout the network. These localized patterns of activity encode the *who* of incoming data.

Again, we first address the analysis of networks receiving a single stimulus. When a neuron receives an external tonic input, this unit increases its bursting frequency (see Section 3.1). This increase can make the neighbor units recognize the neural signature of the stimulated neuron and propagate the corresponding intraburst firing pattern. In this situation, new intriguing collective dynamics arise in the network. To illustrate the dynamic spatial organization of the neural signatures traveling through the network, we generate activity movies representing the fingerprint-based evolving dynamics (see Section 2.2.2 for details). These activity movies point out that the network generates in this dimension well-defined transient patterns of activity in response to data onset. The emerging spatio-temporal patterns are related to the spatial organization and clusterization of the signatures traveling through the network. To give insight into the generation and propagation of these complex spatio-temporal structures, Figure 5 shows snapshots of the activity movies of two representative networks in which the same unit receives an input. Note, that the only signature traveling through the network corresponds to the stimulated unit. If we consider that at a given moment two neurons that recognize the same signature belong to the same cluster; we can study the specific properties of the dynamic organization of the patterns by calculating the clustering coefficient and the average shortest path between neurons belonging to the same cluster. This analysis indicates that the fingerprint-based spatio-temporal patterns are initially originated in the stimulated unit (see initial frames in the sequences of Figure 5). Then, depending on the parameters *M*_*i*_ and *L*_*i*_, they can propagate locally or globally as transient wave fronts; or as localized clusters with a fixed spatial organization that occasionally become the source of new transient patterns. The generation of localized transient patterns of activity in the fingerprint spatio-temporal space suggests a collective coding strategy based on the emission and recognition of specific neural fingerprints. This mechanism allows the network to encode information regarding the origin of incoming data (input source) in a distributed network form.

The information encoded in the spike-timing modality and the encoded in the rhythmic modality coexist in the network. A relevant property observed in the simulations is that a neural fingerprint does not necessarily travel over the propagating wave fronts encoding the corresponding spiking-bursting rhythm. The spreading velocity of the fingerprint-based spatio-temporal patterns is always slower than the corresponding spiking-bursting spatio-temporal patterns velocity (cf. time interval between frames in Figures 3, 5; 33 vs. 1000 *a*.*u*). Likewise, the spatial organization of the patterns in the different spatio-temporal spaces is not correlated. If we consider that at a given moment two neurons over the firing threshold belong to the same cluster, we can calculate the clustering coefficient and the average shortest path for the spiking-bursting patterns and compare the self-organizing properties of the patterns encoded in both information dimension. This analysis points out that the spiking-bursting patterns always consist of propagating transient wave fronts from the stimulated unit traveling through the whole network. Meanwhile, the fingerprint-based patterns can also be originated in the stimulated unit, but they can propagate locally or globally as transient wave fronts or remain bounded in specific regions of the network.

**Figure 6:**
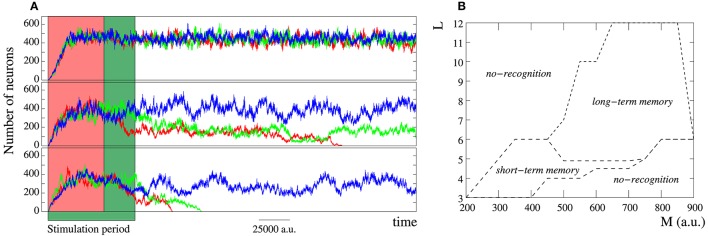
**(A)** Evolution of the mean number of neurons that recognize and emit the fingerprint of a unit receiving the same data in three different networks during three different periods. Each trace is calculated as the average of 10 experiments with different random seeds and location of the stimulated unit. These plots characterize the stimuli-evoked fingerprint-based dynamics. Top panel: *M*_*i*_ = 500 and *L*_*i*_ = 5. Middle panel: *M*_*i*_ = 400 and *L*_*i*_ = 4. Bottom panel: *M*_*i*_ = 350 and *L*_*i*_ = 4. Units are dimensionless. In red traces, the stimulation period corresponds to the red region. In green traces, to the green region. And in blue traces, data are continuously present. In this spatio-temporal space, the network may act as a long-term memory (top panel) or as a short-term memory (middle and bottom panels) depending on the value of *M*_*i*_ and *L*_*i*_, i.e., the parameters associated to intra-unit contextualization. **(B)** Phase diagram illustrating the relationship between *M*_*i*_ and *L*_*i*_ in networks where *p* = 0.05, *TH* = 50, *RP* = 50, and *AP* = 200 (units are dimensionless).

A simple way to characterize the fingerprint-based dynamics is computing the number of neurons that recognize and emit a given firing pattern. This allows us to identify the signatures encoded in the network. Figure 6A depicts the characteristic evolution of the level of activity related to the fingerprint of the data source in three representative networks receiving the same single input during three different stimulation periods. This figure corroborates the results derived from the snapshots shown in Figure 5. When the stimulation begins, the signature of the stimulated unit starts propagating through the network. The number of neurons recognizing this fingerprint grows until reaching a stationary level that depends on the value of *M*_*i*_ and *L*_*i*_. Then, the network dynamics consists of a fluctuation around the steady level (e.g., see blue traces in Figure 6A). This dynamic is kept while the stimulation is sustained. When the stimulation ends, the stimulus-evoked activity does not immediately disappear from the network (cf. red and green traces in Figure 6A). This is an interesting result that demonstrates that intra-unit contextualization can be a mechanism to implement intrinsic memory in the network, giving rise to both short-term and long-term memories. In short-term memories (bottom and middle panel in Figure 6A), the stimuli-evoked dynamics reverberate for a while. This reverberation effect constitutes a mechanism providing the network the ability of acting as a dynamical working memory that transiently stores incoming data. In contrast, in long-term memory networks (top panel in Figure 6A), the information survives in the network in a permanent manner (maybe until a new input is received).

The collective dynamics in the fingerprint-based dimension is mainly driven by the intra-unit parameters *M*_*i*_ and *L*_*i*_. On the one hand, reducing the size of the local informational context of every neuron (*M*_*i*_) decreases the number of neurons that recognize a given firing pattern. On the other hand, decreasing the learning threshold (*L*_*i*_) facilitates the recognition of the propagating fingerprints and, therefore, the level of activity in the network grows. The trade-off among the effect of these parameters determines if the network encodes information in the spike-timing modality and the mode of behavior in this dimension. To illustrate this, Figure 6B depicts a phase diagram locating the different behaviors in the space of intra-unit parameters.

**Figure 7:**
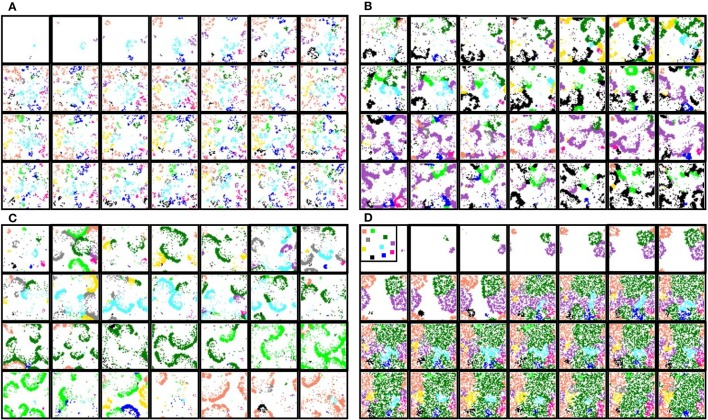
**Snapshots of four representative activity movies illustrating the fingerprint-based spatio-temporal patterns generated by networks that receive 10 data simultaneously**. The inset in the first frame of **(D)** shows the approximate location of each input. Sequences develop in time from left to right and from top to bottom. The time interval between frames is 2000 *a*.*u*. Subcellular plasticity induces different competition dynamics among the coexisting patterns in this spatio-temporal space: from winnerless **(A–C)** to winner-take-all **(D)**. These competition regimes are characterized in Figure 8. **(A)** *p* = 0.05, *M*_*i*_ = 400, and *L*_*i*_ = 4. The competition among fingerprints makes the patterns only propagate locally, remaining bounded near the corresponding stimulated unit. **(B)** *p* = 0.05, *M*_*i*_ = 350, and *L*_*i*_ = 4. Evolving coexisting patterns propagate through the whole ensemble. Each pattern is originated in the unit that receives the corresponding input. **(C)** *p* = 0.05, *M*_*i*_ = 500, and *L*_*i*_ = 5. The patterns also travel through the whole network, but there exist alternating periods during which only the patterns encoding a given input propagate. After that, a new competing cycle begins until a fingerprint prevails over the others and starts propagating. **(D)** *p* = 0.08, *M*_*i*_ = 350, and *L*_*i*_ = 3. As result of the competition, only the patterns associated to a limited group of data (the winners) propagate. Note that the different competition regimes arise depending on the values *M*_*i*_ and *L*_*i*_ which shape the intra-unit contextualization mechanism.

With the experiments described so far, we investigate the ability of the proposed network to encode and process a single stimulus using an information processing strategy driven by local contextualization. If we repeat the same experiments but now introducing multiple inputs simultaneously, we observe that the presence of multiple stimuli makes the network generate coexisting transient spatio-temporal patterns of activity encoding the origin of the different inputs (Figure 7). These experiments reveal additional relevant computational properties that subcellular plasticity can provide to spiking neural networks. When multiple intraburst firing patterns spread through the network, a competition dynamics arises between them. A simple visual inspection of the snapshots shown in Figure 7 reveals that the self-organizing properties of the patterns drastically change depending on the intra-unit parameters shaping the intra-unit plasticity rules. These define different modes of competition among the spreading fingerprints. This competition affects the global level of activity of each signature in the network and determines the spatial organization of the patterns. The competition dynamics among the different intraburst firing patterns determines the coherence and coordination of the coexisting patterns.

We would like to highlight that the competition regimes observed in the activity movies arise in the absence of inhibitory connections, which hints at intra-unit contextualization as an effective mechanism to restrict the activity in networks without inhibition. Note that each neuron can only transmit one recognized firing pattern per burst. This limitation produces somehow a local competition among the patterns received by the neuron where only the “winner” is transmitted. This local competition is the basis of the global competition in the whole network.

**Figure 8:**
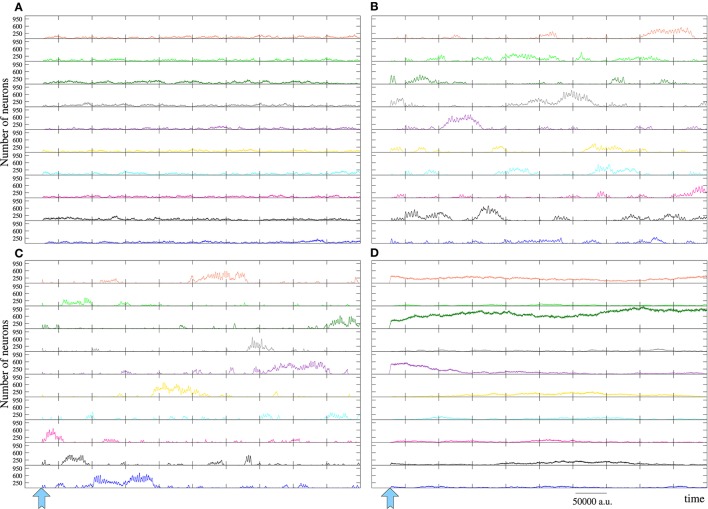
**Level of activity related to the 10 neural signatures belonging to the input sources in the networks of Figure 7**. The inputs and the color code used to identify them are the same used in this figure. All of them are injected simultaneously from time step pointed out by the arrow to the end of the time series. Each panel corresponds to the equivalent in Figure 7 and illustrates a different competition regime (see main text for details).

The different dynamical modes observed in the activity movies are better characterized by the evolution of the number of neurons that recognize and emit each signature (Figure 8). Regardless the number of active inputs, the type of competition depends on the value of the parameters *M*_*i*_ and *L*_*i*_ and may vary from a winnerless (WLC) to a winner-take-all (WTA) competition. In WLC networks, none of the signatures becomes a “winner,” and therefore, none of them persistently prevails over the others. Depending on the intra-unit parameters, the network can display different winnerless regimes. Figure 8A illustrates a winnerless competition in which the level of activity related to every fingerprint is similar and remains fluctuating nearly a stationary level. This defines a collective dynamics where several coherent spatio-temporal patterns coexist within the network encoding simultaneously a great amount of data (e.g., in Figure 8A all the inputs introduced into the network). Figures 8B,C show winnerless regimes with alternating periods where some fingerprint has a higher level of activity.

**Figure 9:**
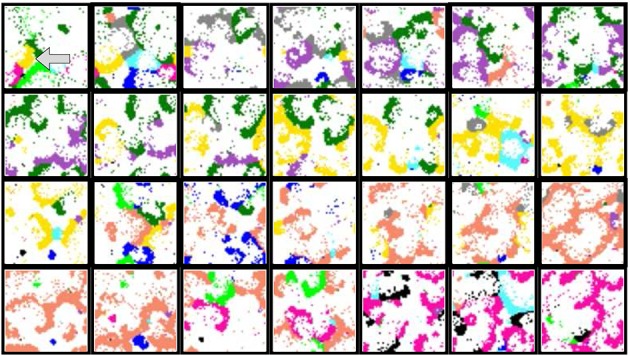
**WLC network whose collective dynamics is characterized by an emitter area that generates transient patterns encoding the prevailing fingerprint in the network**. Arrow in the first frame denotes the approximate location of this area. Sequences develop in time from left to right and from top to bottom. When neurons in the pointed area start generating patterns encoding a given input, the collective behavior changes accordingly to these patterns and the corresponding fingerprint prevails over the others. Note that the existence of these emitter areas is a self-organizing property of the network.

An interesting phenomenon observed with some network settings is that some regions within the network specialize in the emission of firing patterns encoding the origin of different stimuli although they do not receive any external input. This phenomenon occurs without any kind of supervised synaptic nor intra-cellular learning, i.e., it is a self-organizing property of the network. These emitter areas are usually related to winnerless competitions where the prevailing fingerprints change accordingly to the patterns originated in these areas (Figure 9). Conversely, when a winner-take-all competition occurs, only the signature or signatures that win the competition propagates through the network (e.g., see Figure 8D). In the WTA network shown in Figure 7D, all the neurons tend to recognize and emit simultaneously the prevailing fingerprint. However, depending on *M*_*i*_ and *L*_*i*_, this can also spread as evolving transient patterns equivalent to the shown in Figure 7C when the dark green input prevails over the others. Note that, in some sense, the winnerless competitions displayed in Figures 7B,C consist of sequences of transient winner-take-all competitions.

The reverberating spatio-temporal patterns encoding the origin of incoming data continue competing even when they are not sustained by an active input. Short-term memory networks have a limited ability to retain previously stored data when new information is introduced into the network. In these cases, the reverberation period drops as compared to networks receiving a single input, and the stored data are almost instantaneously forgotten, i.e., the corresponding patterns disappear because the patterns encoding the last incoming stimulus win the competition. However, in long-term memory networks, coexisting coherent spatio-temporal patterns related to multiple fingerprints can be observed even when the corresponding input is not active.

## 4. Discussion

The present work introduces a spiking neural network that makes use of multicoding strategies for information propagation and subcellular plasticity to locally contextualize or discriminate data received by a unit. Furthermore, each neuron in the network has a neural signature that allows its unequivocal identification by the rest of the cells. This network is an encoder and generator of spatio-temporal patterns that take advantage of the multiple simultaneous encoding modalities present in the network to transform dynamic inputs into different spatio-temporal spaces, and organize and coordinate coexisting patterns of transient activity in response to data onset.

The discussed experiments are aimed at analyzing the emerging collective dynamics in two information dimensions. On one hand, a spiking-bursting spatio-temporal space, where information processing is driven by synaptic transmission. On the other hand, a fingerprint-based spatio-temporal space driven by an intra-unit contextualization mechanism. The specific properties of the dynamic organization of the patterns are different in each information dimension, so that, the life cycle of the information encoded in both encoding schemes is independent. When multiple patterns in the same dimension coexist in the network, a competition emerges between them. We show that various forms of competition can arise without inhibitory connections in the network. Depending on the parameters shaping simple intra-unit plasticity rules, the competition regime may vary from a winnerless (i.e., the network stores multiple data simultaneously) to a winner-take-all competition (i.e., one datum or a group of them prevails over the others). The stimuli-evoked spatio-temporal patterns and the corresponding competing dynamics can survive for long periods after data onset. This reverberation effect allows the network to memorize incoming data. This can display short-term or long-term memory capabilities in the different spatio-temporal spaces. When the network behaves as a short-term memory, the spatio-temporal patterns encoding incoming data in the corresponding scheme transiently reverberate after the stimulation ending. Conversely, in long-term memories, the stimulus leads the network to a new stable state and the patterns persistently survive. The memory ability of the network in each dimension varies as a function of the synaptic and/or intra-unit parameters. Therefore, different simultaneous processing strategies can be implemented within the network.

These results illustrate the dynamical richness and large flexibility of the proposed network to encode and process information in different spatio-temporal spaces. We argue that plasticity mechanisms inside individual cells and multicoding strategies can provide additional computational properties to spiking neural networks, which could enhance their capacity and performance. In particular, local contextualization mechanisms allow individual neurons to process the multiple simultaneous codes in their input signals selectively or globally in order to completely decide or weight the decision about their output in the different encoding schemes. This information processing provides a framework to model complex high-dimensional processes that can be applied to different real-world computational problems. The ideas relating multicoding with local information discrimination have a direct application in problems that benefit from multifunctionality and parallelism. These are desirable features for many technical applications of ANNs, representing a potential advantage when processing large amounts of data or multiple decision-making criteria must be developed, for instance, in multiobjective optimization problems (Saini and Saraswat, 2013; Wang et al., 2014) or in control systems [e.g., multifunctional prosthesis controllers that must quickly detect and classify multiple characteristic simultaneous myoelectric signals (Saridis and Gootee, 1982; Hudgins et al., 1993; Karlik et al., 2003; Li et al., 2010)]. Another straightforward application of these concepts is in problems where a global task is solved by means of solving independent partial tasks. An example is the wide scope of multidimensional sorting problems, specifically when the order in a particular dimension can be independent of the order in other dimensions, or when there is no global sorting criteria in any dimension. Non-spiking signatures neural networks have been successfully applied to this type of problems (Latorre et al., 2011). Areas of application for multidimensional sorting are scheduling, planning and optimization, between others (Catoni, 1998; Aref and Kamel, 2000). On the other hand, the different dynamical modes observed in the network are relevant in the context of multiple technical applications. Winnerless competition is usually associated to sequential information processing (Seliger et al., 2003; Rabinovich et al., 2006a; Arena et al., 2009; Kiebel et al., 2009; Latorre et al., 2013b), which has a wide application in many artificial intelligent systems in tasks such as inference, planning, reasoning, natural language processing, and others (Sun and Giles, 2001; Wörgötter and Porr, 2005). Similarly, pattern recognition in different spiking ANNs is based on winner-take-all dynamics (Bohte et al., 2002a; Gütig and Sompolinsky, 2006; Schmuker et al., 2014).

In this paper, we have imposed some constraints and assumptions in order to facilitate the presentation of our results. Results obtained with larger regular networks (up to 1000 × 1000); higher levels of bursting activity; and different number and/or distribution of spikes in the neural signatures are equivalent to the results presented in Section 3. In experiments with signatures with an arbitrary number of spikes, new interesting fingerprint-based dynamics emerges in the network and results are not exactly the same. In these simulations, not only the fingerprints belonging to a neuron propagate, but also specific firing sequences built with combinations of these signatures propagate throughout the network. In some sense, these networks do not only encode information regarding the input source, but they also generate new information. It is also important to note that, for simplicity, we only consider two encoding schemes in the network. However, bursting activity allows easily including additional units of information (e.g., the burst duration or the number of spikes in the burst). In this line, and regarding a selective processing of input messages, experimental evidence indicates that some neural systems exhibit *functional or behavioral neural signatures* representing different states or associated to the task performed at a given moment (Klausberger et al., 2003; Somogyi and Klausberger, 2005; Kaping et al., 2011). The concept of neural fingerprint that underlies the strategy of the discussed network can be extended to consider the emission and recognition of multiple fingerprints with a different meaning within the same signal. In this situation, subcellular plasticity in the form of intra-unit information contextualization mechanisms would allow individual neurons to perform a distinct processing of incoming signals, for example, as a function of specific emitters and/or functional states.

Although not addressed in this paper, subcellular plasticity and multicoding mechanisms for information processing can be combined with the features that underlie information processing in the existing spiking neural network paradigms. In this line, for example, plenty of work has been done on synaptic plasticity in spiking neural networks, since modifications of the synaptic connections are traditionally considered the physiological basis of learning in the nervous system. These works are mostly related to unsupervised synaptic learning methods, such as Spike-Timing Dependent Plasticity (STDP) (Song et al., 2000; Bohte et al., 2002b; Kube et al., 2008; Meftah et al., 2010), with an increasing interest into supervised synaptic learning (Bohte et al., 2002a; Belatreche et al., 2007; Yu et al., 2013). The combination of learning rules including not only the modification of the synaptic weights, but also the parameters that affect the local discrimination of input signals can greatly contribute to enhance the spiking ANNs' computational power. In this vein, our results can be of particular interest in the context of the generation and recognition of spatio-temporal information. Different spiking neural networks have been proposed to process, classify, and store spatio-temporal patterns (Laje and Buonomano, 2013; Yu et al., 2013). We speculate that incorporating multicoding strategies and different types of subcellular plasticity to other successful spiking ANN paradigms can potentially allow these networks to process, classify and store more complex data. For example, a highly relevant application of the referred spiking networks is the analysis of EEG spatio-temporal data. To consider a multicoding mechanism that incorporates the neural fingerprint-based dimension to these networks could permit an analysis of coexisting brain rhythms from multiple simultaneous perspectives. In particular, the fingerprint-based spatio-temporal patterns could facilitate the analysis of the propagation trajectories and the identification of possible information sources and sinks in different cognitive processes.

Because of their functional similarity to biological neurons, spiking neural networks have been extensively used by the computational neuroscience community as a powerful tool for studying neural information processing (e.g., see Izhikevich, 2003; Deco et al., 2008; Izhikevich and Edelman, 2008). Results obtained with our simple model could also be relevant from this perspective Information storage in the nervous system has been typically studied considering the adaptation of the synaptic connection strengths (e.g., see Zipser et al., 1993). Our simulations suggest that mechanisms inside individual cells modulating their intrinsic dynamics could also be an effective mechanism to implement intrinsic memory, both in short- and long-term memory networks. On the other hand, many biological neural systems (including many areas of the human brain) continuously receive a great amount of inputs from many different sources and, nevertheless, they exhibit a low level of activity and only respond to specific inputs (Shoham et al., 2006; Sato et al., 2007; O'Connor et al., 2010; Barth and Poulet, 2012). We hypothesize that neural dynamics based on the propagation of specific neural fingerprints and a contextualization mechanisms like the one studied here could explain why these system are so sparsely active. Target neurons would only fire when they recognize a characteristic firing pattern in their incoming stimuli; while signal not recognized would be simply ignored. Obviously, to test this hypothesis more realistic spiking models for the activity of the neurons must be developed.

## Author contributions

RL conceived and designed the study. JC and RL conducted the experiments. JC and RL analyzed the data. JC and RL wrote, read, and approved the manuscript.

## Funding

This work was supported by UAM-Banco Santander (CEAL-AL/2015-16) and MINECO/FEDER DPI2015-65833-P.

### Conflict of interest statement

The authors declare that the research was conducted in the absence of any commercial or financial relationships that could be construed as a potential conflict of interest.

## References

[B1] Aref W. G. Kamel I. (2000). On multi-dimensional sorting orders, in Lecture Notes in Computer Science, Vol. 1873 (Berlin; Heidelberg: Springer), 774–783.

[B2] Arena P. Fortuna L. Lombardo D. Pantanè L. Velarde M. G. (2009). The winnerless competition paradigm in cellular nonlinear networks: models and applications. Int. J. Circ. Theory Appl. 37, 505–528. 10.1002/cta.567

[B3] Barth A. L. Poulet J. F. (2012). Experimental evidence for sparse firing in the neocortex. Trends Neurosci. 35, 345–355. 10.1016/j.tins.2012.03.008 22579264

[B4] Belatreche A. Maguire L. McGinnity M. (2007). Advances in design and application of spiking neural networks. Soft Comput. 11, 239–248. 10.1007/s00500-006-0065-7

[B5] Bialek W. Rieke F. de Ruyter van Steveninck R. R. Warland D. (1991). Reading a neural code. Science 252, 1854–1857. 10.1126/science.2063199 2063199

[B6] Bishop C. M. (1995). Neural Networks for Pattern Recognition. New York, NY: Oxford University Press, Inc.

[B7] Bohte S. M. (2004). The evidence for neural information processing with precise spike-times: a survey. Nat. Comput. 3, 195–206. 10.1023/B:NACO.0000027755.02868.60

[B8] Bohte S. M. La Poutre H. Kok J. N. (2002a). Error-backpropagation in temporally encoded networks of spiking neurons. Neurocomputing 48, 17–37. 10.1016/S0925-2312(01)00658-0

[B9] Bohte S. M. Poutre H. L. Kok J. N. (2002b). Unsupervised clustering with spiking neurons by sparse temporal coding and multilayer rbf networks. IEEE Trans. Neural Netw. 13, 426–435. 10.1109/72.991428 18244443

[B10] Brette R. Rudolph M. Carnevale T. Hines M. Beeman D. Bower J. M.. (2007). Simulation of networks of spiking neurons: a review of tools and strategies. J. Comput. Neurosci. 23, 349–398. 10.1007/s10827-007-0038-6 17629781 PMC2638500

[B11] Brochini L. Carelli P. V. Pinto R. D. (2011). Single synapse information coding in intraburst spike patterns of central pattern generator motor neurons. J. Neurosci. 31, 12297–12306. 10.1523/JNEUROSCI.1568-11.2011 21865472 PMC6623235

[B12] Campos D. Aguirre C. Serrano E. Rodríguez F. B. de Polavieja G. G. Varona P. (2007). Temporal structure in the bursting activity of the leech heartbeat CPG neurons. Neurocomputing 70, 1792–1796. 10.1016/j.neucom.2006.10.118

[B13] Carrillo-Medina J. L. Latorre R. (2015). Neural dynamics based on the recognition of neural fingerprints. Front. Comput. Neurosci. 9:33. 10.3389/fncom.2015.00033 25852531 PMC4371706

[B14] Catoni O. (1998). Solving scheduling problems by simulated annealing. Siam J. Control Optim. 36, 1539–1575. 10.1137/S0363012996307813

[B15] Cessac B. Paugam-Moisy H. Viéville T. (2010). Overview of facts and issues about neural coding by spikes. J. Physiol. Paris 104, 5–18. 10.1016/j.jphysparis.2009.11.002 19925865

[B16] Davis G. W. (2006). Homeostatic control of neural activity: from phenomenology to molecular design. Annu. Rev. Neurosci. 29, 307–323. 10.1146/annurev.neuro.28.061604.135751 16776588

[B17] Deco G. Jirsa V. K. Robinson P. A. Breakspear M. Friston K. (2008). The dynamic brain: from spiking neurons to neural masses and cortical fields. PLoS Comput. Biol. 4:e1000092. 10.1371/journal.pcbi.1000092 18769680 PMC2519166

[B18] Deco G. Schürmann B. (1998). The coding of information by spiking neurons: an analytical study. Network 9, 303–317. 10.1088/0954-898X_9_3_002 9861992

[B19] Diehl P. U. Neil D. Binas J. Cook M. Liu S.-C. Pfeiffer M. (2015). Fast-classifying, high-accuracy spiking deep networks through weight and threshold balancing, in 2015 International Joint Conference on Neural Networks (IJCNN) (Killarney), 10.1109/IJCNN.2015.7280696

[B20] Diesmann M. Gewaltig M. O. Aertsen A. (1999). Stable propagation of synchronous spiking in cortical neural networks. Nature 402, 529–533. 10.1038/990101 10591212

[B21] Elson R. C. Huerta R. Abarbanel H. D. Rabinovich M. I. Selverston A. I. (1999). Dynamic control of irregular bursting in an identified neuron of an oscillatory circuit. J. Neurophysiol. 82, 115–122. 10400940 10.1152/jn.1999.82.1.115

[B22] Esser S. K. Merolla P. A. Arthur J. V. Cassidy A. S. Appuswamy R. Andreopoulos A.. (2016). Convolutional networks for fast, energy-efficient neuromorphic computing. Proc. Natl. Acad. Sci. U.S.A. 113, 11441–11446. 10.1073/pnas.1604850113 27651489 PMC5068316

[B23] Garcia L. D'Alessandro G. Fernagut P.-O. Bioulac B. Hammond C. (2005). Impact of high-frequency stimulation parameters on the pattern of discharge of subthalamic neurons. J. Neurophysiol. 94, 3662–3669. 10.1152/jn.00496.2005 16148275

[B24] Gerstner W. (1995). Time structure of the activity in neural network models. Phys. Rev. E Stat. Phys. Plasmas Fluids Relat. Interdiscip. Topics 51, 738–758. 10.1103/PhysRevE.51.738 9962697

[B25] Gerstner W. Kistler W. (2002). Spiking Neuron Models: Single Neurons, Populations, Plasticity. Cambridge, MA: Cambridge University Press 10.1017/cbo9780511815706

[B26] Gerstner W. Ritz R. van Hemmen J. L. (1993). Why spikes? Hebbian learning and retrieval of time-resolved excitation patterns. Biol. Cybern. 69, 503–515. 10.1007/BF00199450 7903867

[B27] Gütig R. Sompolinsky H. (2006). The tempotron: a neuron that learns spike timing-based decisions. Nat. Neurosci. 9, 420–428. 10.1038/nn1643 16474393

[B28] Hindmarsh J. L. Rose R. M. (1984). A model of neuronal bursting using three coupled first order differential equations. Proc. Roy. Soc. B Biol. Sci. 221, 87–102. 10.1098/rspb.1984.0024 6144106

[B29] Hudgins B. Parker P. Scott R. (1993). A new strategy for multifunction myoelectric control. IEEE Trans. Biomed. Eng. 40, 82–94. 10.1109/10.204774 8468080

[B30] Izhikevich E. (2006). Dynamical Systems in Neuroscience: The Geometry of Excitability and Bursting. Cambridge, MA: MIT Press.

[B31] Izhikevich E. M. (2003). Simple model of spiking neurons. IEEE Trans. Neural Netw. 14, 1569–1572. 10.1109/TNN.2003.820440 18244602

[B32] Izhikevich E. M. (2004). Which model to use for cortical spiking neurons? IEEE Trans. Neural Netw. 15, 1063–1070. 10.1109/TNN.2004.832719 15484883

[B33] Izhikevich E. M. Edelman G. M. (2008). Large-scale model of mammalian thalamocortical systems. Proc. Natl. Acad. Sci. U.S.A. 105, 3593–3598. 10.1073/pnas.0712231105 18292226 PMC2265160

[B34] Kampakis S. (2013). Investigating the computational power of spiking neurons with non-standard behaviors. Neural Netw. 43C, 41–54. 10.1016/j.neunet.2013.01.011 23500499

[B35] Kandel E. R. Schwartz J. Jessell T. M. (eds.). (1991). Principles of Neural Science, 3rd Edn. New York, NY: Elsevier Science Publishing Co. Inc.

[B36] Kaping D. Vinck M. Hutchison R. M. Everling S. Womelsdorf T. (2011). Specific contributions of ventromedial, anterior cingulate, and lateral prefrontal cortex for attentional selection and stimulus valuation. PLoS Biol. 9:e1001224. 10.1371/journal.pbio.1001224 22215982 PMC3246452

[B37] Karlik B. Tokhi M. O. Alci M. (2003). A fuzzy clustering neural network architecture for multifunction upper-limb prosthesis. IEEE Trans. Biomed. Eng. 50, 1255–1261. 10.1109/TBME.2003.818469 14619995

[B38] Kayser C. Montemurro M. A. Logothetis N. K. Panzeri S. (2009). Spike-phase coding boosts and stabilizes information carried by spatial and temporal spike patterns. Neuron 61, 597–608. 10.1016/j.neuron.2009.01.008 19249279

[B39] Kepecs A. Lisman J. (2003). Information encoding and computation with spikes and bursts. Network 14, 103–118. 10.1080/net.14.1.103.118 12613553

[B40] Kepecs A. Lisman J. (2004). How to read a burst duration code. Neurocomputing 58–60, 1–6. 10.1016/j.neucom.2004.01.014

[B41] Kiebel S. J. von Kriegstein K. Daunizeau J. Friston K. J. (2009). Recognizing sequences of sequences. PLoS Comput. Biol. 5:e1000464. 10.1371/journal.pcbi.1000464 19680429 PMC2714976

[B42] Klausberger T. Magill P. J. Márton L. F. Roberts J. D. B. Cobden P. M. Buzsáki G.. (2003). Brain-state- and cell-type-specific firing of hippocampal interneurons *in vivo*. Nature 421, 844–848. 10.1038/nature01374 12594513

[B43] Komendantov A. O. Kononenko N. I. (1996). Deterministic chaos in mathematical model of pacemaker activity in bursting neurons of snail, helix pomatia. J. Theor. Biol. 183, 219–230. 10.1006/jtbi.1996.0215 8977879

[B44] Kube K. Herzog A. Michaelis B. de Lima A. D. Voigt T. (2008). Spike-timing-dependent plasticity in small-world networks. Neurocomputing 71, 1694–1704. 10.1016/j.neucom.2007.03.013

[B45] Laje R. Buonomano D. V. (2013). Robust timing and motor patterns by taming chaos in recurrent neural networks. Nat. Neurosci. 16, 925–933. 10.1038/nn.3405 23708144 PMC3753043

[B46] Latorre R. Aguirre C. Rabinovich M. I. Varona P. (2013a). Transient dynamics and rhythm coordination of inferior olive spatio-temporal patterns. Front. Neural Circuits 7:138. 10.3389/fncir.2013.00138 24046731 PMC3763220

[B47] Latorre R. Levi R. Varona P. (2013b). Transformation of context-dependent sensory dynamics into motor behavior. PLoS Comput. Biol. 9:e1002908. 10.1371/journal.pcbi.1002908 23459114 PMC3572992

[B48] Latorre R. Rodríguez F. B. Varona P. (2002). Characterization of triphasic rhythms in central pattern generators (i): interspike interval analysis, in Lecture Notes in Computer Science (Berlin; Heidelberg: Springer), 160–166. 10.1007/3-540-46084-5_27

[B49] Latorre R. Rodríguez F. B. Varona P. (2004). Effect of individual spiking activity on rhythm generation of central pattern generators. Neurocomputing 58, 535–540. 10.1016/j.neucom.2004.01.091

[B50] Latorre R. Rodríguez F. B. Varona P. (2006). Neural signatures: multiple coding in spiking-bursting cells. Biol. Cybern. 95, 169–183. 10.1007/s00422-006-0077-5 16830138

[B51] Latorre R. Rodríguez F. B. Varona P. (2007). Reaction to neural signatures through excitatory synapses in central pattern generator models. Neurocomputing 70, 1797–1801. 10.1016/j.neucom.2006.10.059

[B52] Latorre R. Rodríguez F. B. Varona P. (2011). Signature neural networks: definition and application to multidimensional sorting problems. IEEE Trans. Neural Netw. 22, 8–23. 10.1109/TNN.2010.2060495 21095867

[B53] Lestienne R. (1996). Determination of the precision of spike timing in the visual cortex of anaesthetised cats. Biol. Cybern. 74, 55–61. 10.1007/BF00199137 8573653

[B54] Li G. Schultz A. E. Kuiken T. A. (2010). Quantifying pattern recognition-based myoelectric control of multifunctional transradial prostheses. EEE Trans. Neural Syst. Rehabil. Eng. 18, 185–192. 10.1109/TNSRE.2009.2039619 20071269 PMC3024915

[B55] Liu A. Golowasch J. Marder E. Abbott F. (1998). A model neuron with activity-dependent conductances regulated by multiple calcium sensor. J. Neurosci. 18, 2309–2320. 9502792 10.1523/JNEUROSCI.18-07-02309.1998 PMC6793093

[B56] Maass W. (1996). Noisy spiking neurons with temporal coding have more computational power than sigmoidal neurons, in Advances in Neural Information Processing Systems 9, NIPS, eds Mozer M. Jordan M. I. Petsche T. (Denver, CO: MIT Press), 211–217.

[B57] Maass W. (1997a). Fast sigmoidal networks via spiking neurons. Neural Comput. 9, 279–304. 10.1162/neco.1997.9.2.279 9117904

[B58] Maass W. (1997b). Networks of spiking neurons: the third generation of neural network models. Neural Netw. 10, 1659–1671. 10.1016/S0893-6080(97)00011-7

[B59] Maass W. Bishop C. M. (2001). Pulsed Neural Networks. Cambridge, MA: MIT Press.

[B60] Mainen Z. F. Sejnowski T. J. (1995). Reliability of spike timing in neocortical neurons. Science 268, 1503–1506. 10.1126/science.7770778 7770778

[B61] Mallat S. (1999). A Wavelet Tour of Signal Processing. San Diego, CA; London: Academic Press.

[B62] Marin B. Pinto R. D. Elson R. C. Colli E. (2014). Noise, transient dynamics, and the generation of realistic interspike interval variation in square-wave burster neurons. Phys. Rev. E 90:042718. 10.1103/physreve.90.042718 25375534

[B63] Meftah B. Lezoray O. Benyettou A. (2010). Segmentation and edge detection based on spiking neural network model. Neural Process. Lett. 32, 131–146. 10.1007/s11063-010-9149-6

[B64] Michie D. Spiegelhalter D. J. Taylor C. C. Campbell J. (eds.). (1994). Machine Learning, Neural and Statistical Classification. Upper Saddle River, NJ: Ellis Horwood.

[B65] Middleton J. W. Yu N. Longtin A. Maler L. (2011). Routing the flow of sensory signals using plastic responses to bursts and isolated spikes: experiment and theory. J. Neurosci. 31, 2461–2473. 10.1523/JNEUROSCI.4672-10.2011 21325513 PMC6623695

[B66] Natschläger T. Ruf B. (1998). Spatial and temporal pattern analysis via spiking neurons. Network 9, 319–332. 10.1088/0954-898X_9_3_003 9861993

[B67] O'Connor D. H. Peron S. P. Huber D. Svoboda K. (2010). Neural activity in barrel cortex underlying vibrissa-based object localization in mice. Neuron 67, 1048–1061. 10.1016/j.neuron.2010.08.026 20869600

[B68] O'Connor P. Neil D. Liu S.-C. Delbruck T. Pfeiffer M. (2013). Real-time classification and sensor fusion with a spiking deep belief network. Front. Neurosci. 7:178. 10.3389/fnins.2013.00178 24115919 PMC3792559

[B69] Panzeri S. Brunel N. Logothetis N. K. Kayser C. (2010). Sensory neural codes using multiplexed temporal scales. Trends Neurosci. 33, 111–120. 10.1016/j.tins.2009.12.001 20045201

[B70] Ponulak F. Kasinski A. (2011). Introduction to spiking neural networks: information processing, learning and applications. Acta Neurobiol. Exp. (Wars) 71, 409–433. 22237491 10.55782/ane-2011-1862

[B71] Rabinovich M. I. Huerta R. Varona P. Afraimovich V. S. (2006a). Generation and reshaping of sequences in neural systems. Biol. Cybern. 95, 519–536. 10.1007/s00422-006-0121-5 17136380

[B72] Rabinovich M. I. Varona P. Selverston A. I. Abarbanel H. D. I. (2006b). Dynamical principles in neuroscience. Rev. Mod. Phys. 78, 1213–1265. 10.1103/RevModPhys.78.1213

[B73] Reinagel P. Reid R. C. (2002). Precise firing events are conserved across neurons. J. Neurosci. 22, 6837–6841. Available online at: http://www.jneurosci.org/content/22/16/6837.abstract 12177180 10.1523/JNEUROSCI.22-16-06837.2002 PMC6757890

[B74] Rieke F. Warland D. de Ruyter van Steveninck R. Bialek W. (1999). Spikes: Exploring the Neural Code. Cambridge, MA: MIT Press.

[B75] Rodríguez F. B. Latorre R. Varona P. (2002). Characterization of triphasic rhythms in central pattern generators (ii): Burst information analysis, in Lecture Notes in Computer Science (Berlin; Heidelberg: Springer), 167–173. 10.1007/3-540-46084-5_28

[B76] Ruf B. Schmitt M. (1998). Self-organization of spiking neurons using action potential timing. IEEE Trans. Neural Netw. Berlin; Heidelberg: Springer 9, 575–578. 10.1109/72.668899 18252481

[B77] Rumbell T. Denham S. L. Wennekers T. (2014). A spiking self-organizing map combining STDP, oscillations, and continuous learning. IEEE Trans. Neural Netw. Learn. Syst. 25, 894–907. 10.1109/TNNLS.2013.2283140 24808036

[B78] Saini A. Saraswat A. (2013). Multi-objective day-ahead localized reactive power market clearing model using {HFMOEA}. Int. J. Electr. Power Energy Syst. 46, 376–391. 10.1016/j.ijepes.2012.10.018

[B79] Saridis G. N. Gootee T. P. (1982). EMG pattern analysis and classification for a prosthetic arm. IEEE Trans. Biomed. Eng. 29, 403–412. 10.1109/TBME.1982.324954 7106790

[B80] Sato T. R. Gray N. W. Mainen Z. F. Svoboda K. (2007). The functional microarchitecture of the mouse barrel cortex. PLoS Biol. 5:e189. 10.1371/journal.pbio.0050189 17622195 PMC1914403

[B81] Schmuker M. Pfeil T. Nawrot M. P. (2014). A neuromorphic network for generic multivariate data classification. Proc. Natl. Acad. Sci. U.S.A. 111, 2081–2086. 10.1073/pnas.1303053111 24469794 PMC3926020

[B82] Seliger P. Tsimring L. S. Rabinovich M. I. (2003). Dynamics-based sequential memory: winnerless competition of patterns. Phys. Rev. E Stat. Nonlin. Soft. Matter Phys. 67(1 Pt 1):011905. 10.1103/PhysRevE.67.011905 12636530

[B83] Shoham S. O'Connor D. H. Segev R. (2006). How silent is the brain: is there a dark matter problem in neuroscience? J. Compar. Physiol. A 192, 777–784. 10.1007/s00359-006-0117-6 16550391

[B84] Somogyi P. Klausberger T. (2005). Defined types of cortical interneurone structure space and spike timing in the hippocampus. J. Physiol. 562(Pt 1), 9–26. 15539390 10.1113/jphysiol.2004.078915 PMC1665488

[B85] Song S. Miller K. D. Abbott L. F. (2000). Competitive hebbian learning through spike-timing-dependent synaptic plasticity. Nat. Neurosci. 3, 919–926. 10966623 10.1038/78829

[B86] Stollnitz E. DeRose T. Salesin D. (1996). Wavelets for Computer Graphics: Theory and Applications. San Francisco, CA: Morgan Kaufmann Publishers Inc.

[B87] Sun R. Giles C. (2001). Sequence learning: from recognition and prediction to sequential decision making. IEEE Intell. Syst. 16, 67–70. 10.1109/MIS.2001.1463065

[B88] Szücs A. Abarbanel H. D. Rabinovich M. I. Selverston A. I. (2005). Dopamine modulation of spike dynamics in bursting neurons. Eur. J. Neurosci. 21, 763–772. 10.1111/j.1460-9568.2005.03894.x 15733094

[B89] Szücs A. Pinto R. D. Rabinovich M. I. Abarbanel H. D. Selverston A. I. (2003). Synaptic modulation of the interspike interval signatures of bursting pyloric neurons. J. Neurophysiol. 89, 1363–1377. 10.1152/jn.00732.2002 12626616

[B90] Tabak J. Mascagni M. Bertram R. (2010). Mechanism for the universal pattern of activity in developing neuronal networks. J. Neurophysiol. 103, 2208–2221. 10.1152/jn.00857.2009 20164396 PMC2853287

[B91] Tristán A. Rodríguez F. B. Serrano E. Varona P. (2004). Networks of neurons that emit and recognize signatures. Neurocomputing 58–60, 41–46. 10.1016/j.neucom.2004.01.020

[B92] Turrigiano G. (2007). Homeostatic signaling: the positive side of negative feedback. Curr. Opin. Neurobiol. 17, 318–324. 10.1016/j.conb.2007.04.004 17451937

[B93] Turrigiano G. G. Nelson S. B. (2004). Homeostatic plasticity in the developing nervous system. Nat. Rev. Neurosci. 5, 97–107. 10.1038/nrn1327 14735113

[B94] VanRullen R. Guyonneau R. Thorpe S. J. (2005). Spike times make sense. Trends Neurosci. 28, 1–4. 10.1016/j.tins.2004.10.010 15626490

[B95] Varona P. Torres J. J. Huerta R. Abarbanel H. D. Rabinovich M. I. (2001a). Regularization mechanisms of spiking–bursting neurons. Neural Netw. 14, 865–875. 10.1016/S0893-6080(01)00046-6 11665777

[B96] Varona P. Torres J. J. Abarbanel H. D. Rabinovich M. I. Elson R. C. (2001b). Dynamics of two electrically coupled chaotic neurons: experimental observations and model analysis. Biol. Cybern. 84, 91–101. 10.1007/s004220000198 11205354

[B97] Wang P. Zhu H. Wilamowska-Korsak M. Bi Z. Li L. (2014). Determination of weights for multiobjective decision making or machine learning. IEEE Syst. J. 8, 63–72. 10.1109/JSYST.2013.2265663

[B98] Wiedemann U. A. Lüthi A. (2003). Timing of network synchronization by refractory mechanisms. J. Neurophysiol. 90, 3902–3911. 10.1152/jn.00284.2003 12930814

[B99] Wörgötter F. Porr B. (2005). Temporal sequence learning, prediction, and control: a review of different models and their relation to biological mechanisms. Neural Comput. 17, 245–319. 10.1162/0899766053011555 15720770

[B100] Yu Q. Tang H. Tan K. C. Li H. (2013). Precise-spike-driven synaptic plasticity: learning hetero-association of spatiotemporal spike patterns. PLoS ONE 8:e78318. 10.1371/journal.pone.0078318 24223789 PMC3818323

[B101] Zeck G. M. Masland R. H. (2007). Spike train signatures of retinal ganglion cell types. Eur. J. Neurosci. 26, 367–380. 10.1111/j.1460-9568.2007.05670.x 17650112

[B102] Zhang W. Linden D. J. (2003). The other side of the engram: experience-driven changes in neuronal intrinsic excitability. Nat. Rev. Neurosci. 4, 885–900. 10.1038/nrn1248 14595400

[B103] Zipser D. Kehoe B. Littlewort G. Fuster J. (1993). A spiking network model of short-term active memory. J. Neurosci. 13, 3406–3420. 8340815 10.1523/JNEUROSCI.13-08-03406.1993 PMC6576542

